# Factors associated with malaria infection in Mudzi District, Mashonaland East Zimbabwe, 2019: a case-control study

**DOI:** 10.1186/s12889-020-09872-2

**Published:** 2020-11-19

**Authors:** T. T. Masango, T. K. Nyadzayo, N. T. Gombe, T. P. Juru, G. Shambira, S. Chiwanda, M. T. Tshimanga

**Affiliations:** 1 Department of Community Medicine, University of Zimbabwe, Harare, Zimbabwe; 2 Ministry of Health and Child Care, Family Health Department Nutrition, Harare, Zimbabwe

**Keywords:** Malaria, Outbreak, Case-control, Mudzi

## Abstract

**Background:**

Kondo Rural Health Centre recorded 27 malaria patients between the 27th of January 2019 and the 2nd of February 2019 against an epidemic threshold of 19 with the malaria outbreak being confirmed on the 5th of February 2019. Indoor residual spraying as part of integrated vector management control activities had been done in the district before the onset of the rainy season as well as social behaviour change communication but residents were contracting malaria. We, therefore, investigated the risk factors associated with this outbreak to recommend scientifically effective prevention and control measures.

**Methods:**

We conducted a 1:1 unmatched case-control study. A case was a resident of Mudzi from the 4th of February 2019 who had a positive rapid diagnostic test for malaria randomly selected from the clinic’s line list whilst controls were randomly selected from the neighbourhood of cases. Pretested interviewer-administered questionnaires were used to collect information on demographic characteristics, knowledge and practices of residents in malaria prevention. Data were analysed using Epi info 7.

**Results:**

A total of 567 confirmed malaria cases was recorded with an overall attack rate of 71.7 per 1000 population. Sixty-three case-control pairs were interviewed. The majority of cases 78% (49/63) were from Makaza, Chanetsa and Nyarongo villages which are within 3 km from Vhombodzi dam. A stagnant water body near a house [aOR = 8.0, 95%CI = (2.3–28.6)], engaging in outdoor activities before dawn or after dusk [aOR = 8.3, 95%CI = (1.1–62.7)] and having a house with open eaves [aOR = 5.4, 95%CI = (1.2–23.3)] were independent risk factors associated with contracting malaria. Wearing long-sleeved clothes when outdoors at night [aOR = 0.2, 95%CI = (0.1–0.4)] was protective.

**Conclusion:**

A stagnant water pool close to the homestead and engaging in outdoor activities before dawn and after dusk were modifiable risk factors associated with the malaria outbreak despite the community being knowledgeable on the transmission and prevention of malaria. Community sensitisation and mobilisation in the destruction of stagnant water bodies and cutting of tall grass around homesteads were recommended measures to contain the outbreak.

**Supplementary Information:**

The online version contains supplementary material available at 10.1186/s12889-020-09872-2.

## Background

Malaria is the 4th leading cause of preventable morbidity and mortality in Africa, caused by the protozoan parasite *Plasmodium* species namely *P.falciparum*, *P.malariae*, *P.ovale*, *P.vivax* and *P.knowlesi* [1]. It is spread by the bite of an infective female *Anopheles* mosquito which bites mainly between dusk and dawn [2]. The disease can cause fever, chills, and flu-like illness and if not treated early, it can cause severe complications and death [3]. Young children under 5 years of age and pregnant women are at high risk of infection with malaria [4].

In 2017, two hundred and nineteen million cases of malaria occurred worldwide and 435,000 deaths were reported, mostly children in the African Region [5]. The World Health Organisation’s African Region (WHO/AFRO) had a high contribution to this global malaria burden having contributed 92% of cases and 93% of deaths [6]. Global efforts and investments to fight and contain malaria have led to a remarkable reduction in morbidity and mortality in the last decade with the successful elimination of malaria in eight WHO member countries [5]. Malaria surveillance in endemic regions assists in reducing the burden of morbidity and mortality by providing data for trend analysis, resource allocation, vector control monitoring, drug efficacy and resistance and epidemic detection, preparedness and response [7]. Poor epidemic preparedness and response (EPR) can result in the inability to analyse and interpret surveillance data which leads to late notification and delayed outbreak investigation [8].

Zimbabwe has seasonal and geographic variation in malaria transmission that corresponds closely with the country’s rainfall pattern. In general, the major malaria transmission season occurs during the rainy season between November and April, with the average temperature ranging between 18 and 30 degrees Celsius [9]. *Plasmodium falciparum* has been the predominant malaria parasite causing 98% of all reported malaria cases [10].

Mudzi District in Mashonaland East Province is malaria endemic with some areas traditionally known to be malaria burdened whilst others are almost malaria-free. A review of the weekly disease surveillance data showed that the number of malaria cases with a positive rapid diagnostic test (RDT) at Kondo Rural Health Centre had been on the increase from the 27th of January 2019 with the number of cases almost double the action threshold between the 10th and the 16th of February 2019. There are five villages around Kondo Rural Health Centre with three village health workers assisting in the delivery and promotion of health services. Indoor residual spraying as part of integrated vector management control activities had been done in the district before the onset of the rainy season as well as social behaviour change communication however residents were contracting malaria. This prompted the District Medical Officer to request the Zimbabwe Field Epidemiology Training Program to investigate the malaria outbreak to recommend scientifically effective prevention and control measures. Understanding and documenting the reasons for malaria outbreaks in areas where integrated vector control activities have been done is necessary for the global fight to eliminate morbidity and mortality from malaria. We, therefore, conducted a case-control study to determine the risk factors associated with contracting malaria in this outbreak.

## Methods

### Study setting

Mudzi District in Mashonaland East Province of Zimbabwe has an estimated population of 1,366,522 people according to the 2017 intercensal demographic survey with 88.8% of the population being rural [11]. Agriculture and mining are the main economic activities with 44% of persons aged 15 years and above economically active. The district has a seasonal burden of malaria with most of the transmission during the wet season and peaking between February and April.

### Descriptive epidemiology

Following an invitation from the District Medical Officer to assist in the outbreak, the District Health Executive was interviewed as the key informants to obtain an overview of the outbreak. Surveillance data, line lists and health records were then reviewed to confirm the outbreak. Active case search and environmental assessments were conducted in the five villages around Kondo Rural Health Centre. Using the generated data, an epidemic curve was constructed and the outbreak characterised by time, place and person.

### Study design

Following the descriptive analysis, it was hypothesised that living close to tall uncut grass or open water bodies was a risk factor associated with contracting malaria. A 1:1 unmatched case-control study was then conducted to test this hypothesis. A case was defined as any person residing in Mudzi District for more than 2 weeks from the 4th of February 2019 and who presented with the onset of any of the following signs and symptoms; fever, headache, muscles and joint pains, chills, body weakness and had a positive rapid diagnostic test (RDT) for malaria. A control was defined as any person living in Mudzi District during the same period that did not develop any of the symptoms of malaria.

### Inclusion criteria

#### Cases

Any resident of Mudzi District during the outbreak period, with a positive RDT who consented to participate in the study.

#### Controls

Any resident of Mudzi District during the outbreak period, who consented to participate in the study and had a negative RDT diagnosis of malaria.

### Exclusion criteria

Individuals who had symptoms suggestive of malaria without RDT confirmation of diagnosis were excluded from the study. Any child below 16 years of age were parental or guardian consent for participation was not readily available or denied.

### Sample size

Using Stat Calc function of Epi Info™ 7, assuming staying within 3 km from a river, swamp, dam or stream a significant risk factor for contracting malaria with an odds ratio of 2.7, with 43% of controls having been exposed (study by Kureya et al.: Malaria Outbreak Investigation in Chipinge, Zimbabwe: A Case-Control Study) using a power of 80% and confidence interval of 95% —The calculated minimum sample size was 74 cases and 74 controls.

### Sampling

A line list of malaria cases at Kondo Rural Health Centre was obtained and using numbers assigned to each case, we selected 74 cases using excel generated random numbers. Village health workers were utilised to locate the cases from the respective villages of residence. Controls were randomly selected from the neighbourhood of cases provided they had no clinical diagnosis of malaria.

### Data collection

A pre-tested interviewer-administered questionnaire (supplementary material 1) was used to collect information on demographic characteristics, knowledge and practices of residents in malaria prevention. Most of the questions were adapted from literature and modified to relate to the study’s objectives. Knowledge about malaria was assessed by asking study participants about signs and symptoms of malaria and methods of malaria prevention. Knowledge was then rated using a 5-point Likert scale. Checklists (adopted from updated IDSR guidelines) were used to gather information on the availability of resources to contain the outbreak. Medical records were reviewed to ascertain case management.

### Data analysis

Data were captured and analysed using Epi info 7™. Data were manually checked for completeness, duplication and omission before analysis. After validation of entered data, frequencies, means and proportions were generated. Bivariate analysis was then carried out with calculation of odds ratios, their confidence intervals and *p*-values to identify risk and protective factors that were associated with contracting malaria. Stratified analysis was done to check for confounding and effect modification. Stepwise forward logistic regression was done for all variables that were significantly associated with contracting malaria at the *p* ≤ 0.25 level on bivariate analysis to determine the independent factors associated with contracting malaria.

### Environmental assessment

A walk-through assessment of housing infrastructure, areas surrounding homes and places where cases and controls spend the majority of their time was done to identify mosquito breeding sites within the community. CDC light trapping and larval dipping were used to collect mosquito and larvae samples which were sent to the laboratory for larvae rearing and adult mosquito identification. Observations were done to see if houses had open eaves and if there was tall uncut grass around the homesteads and stagnant water pools within 200 m from the homestead. Assessments were also done for the availability of mosquito nets, the utilisation of repellents and confirmation of indoor residual spraying of homesteads before the onset of the rainy season.

### Permission and ethical considerations

Written informed consent was obtained from participants before interviews which were done privately. Written parental or guardian consent was obtained on behalf of all participants under 16 years of age as well as the child’s verbal assent to participate in the study. Confidentiality was maintained throughout the study by not recording participant names on questionnaires.

## Results

**Fig. 1:**
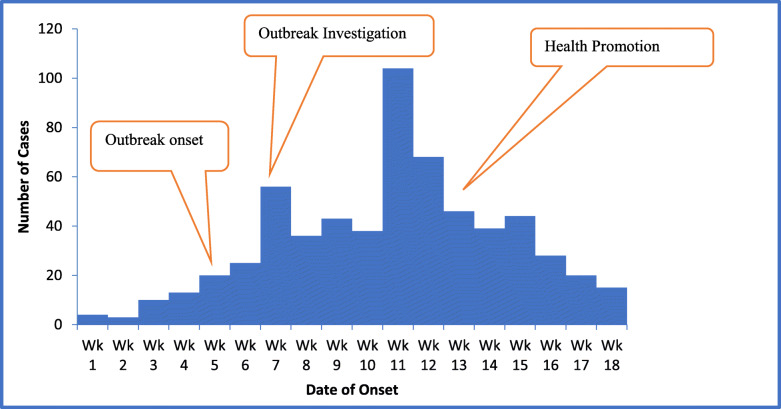
Epidemic curve of malaria outbreak in Mudzi district, Zimbabwe 2019

A total of 567 RDT confirmed malaria case-patients were recorded over the outbreak from week 5 to week 17 of 2019 in the five villages around Kondo Rural Health Centre. The overall attack rate was 71.7 cases per 1000 population (Table 1). Among the malaria cases, 14% (79/567) were from Chanetsa village, 30% (170/567) were from Makaza village, 10% (57/567) were from Vhombodzi village, 13% (74/567) were from Zondo village and 33% (187/567) from Nyarongo village. The greatest number of cases were recorded in week 11 of 2019 and the epidemic curve demonstrated a propagated outbreak (Fig. 1).

**Table 1:** Malaria attack rate by village in Mudzi district, Zimbabwe, 2019

Village	Population	Number of Cases	Attack Rate/1000 population
Chanetsa	1359	79	58.1
Makaza	1945	170	87.4
Vhombodzi	1220	57	46.7
Zondo	1129	74	65.5
Nyarongo	2250	187	83.1
Total	**7903**	**567**	**71.7**

### Characteristics of study participants

Sixty-three case-control pairs out of a target of 74 were recruited giving an 85% response rate. The majority of the cases were male at 60% (38/63) compared to 51% (32/63) females in the controls. Sixty percent of cases (38/63) were not employed compared to 68% (43/63) for controls. The highest level of education attained was primary school attendance by both cases and controls at 59% (37/63) and 55% (35/63) respectively. The median age for cases was 35 years and 31 years for controls. There were no statistically significant differences among cases and controls on the variables: sex, employment status, level of education, religion, age and marital status. Therefore, the socio-demographic characteristics of cases and controls were comparable (Table 2).

**Table 2:** Socio demographic characteristics of respondents in Mudzi district, Zimbabwe 2019

Variable	Category	Cases *n* = 63 (%)	Controls *n* = 63 (%)	*P* Value
Sex	Male	38 (60)	31 (49)	0.28
	Female	25 (40)	32 (51)	
Employment Status	Casual labourer	17 (27)	13 (21)	0.46
	Unemployed	46 (73)	50 (79)	
Marital Status	Single/Minor	13 (21)	12 (19)	0.22
	Married	50 (79)	51 (81)	
Highest level of Education	None	11 (17)	8 (13)	0.54
	Primary	37 (59)	35 (55)	
	Secondary	15 (24)	20 (32)	
Religion	Apostolic	43 (68)	36 (57)	0.41
	Other	20 (32)	27 (43)	
Age	< 5 years	2 (3)	3 (5)	0.61
	5–40 years	52 (83)	48 (76)	
	> 40 years	9 (14)	12 (19)	

### Participants knowledge of malaria

Twenty-two percent (14/63) of cases incorrectly stated infected fruits as the cause of malaria compared to 13% (8/63) of controls. Sixty percent (38/63) of cases managed to recall at least 3 signs and symptoms of malaria compared to 67% (42/63) of controls. The most stated symptom among the cases and controls was fever at 83% (52/63) and 79% (50/63) respectively. The most stated malaria prevention method was sleeping under a treated net 67% (42/63) among cases and 70% (44/63) among controls. Both cases and controls had a fair knowledge of malaria.

### Clinical management of malaria cases

The most common symptoms amongst the malaria cases were fever 87% (55/63), body weakness 78% (49/63), shivering/chills 67% (42/63) and vomiting 56% (35/63). The majority of the cases were diagnosed by health workers at the clinic 71% (45/63) whilst the remainder 29% (18/63) were diagnosed and treated by their respective village health workers. Malaria confirmation in all cases was done using malaria rapid diagnostic tests and any case confirmed positive was put on a 6 dose course of artemether-lumefantrine (1.5 mg/12 mg/kg) according to national treatment guidelines [12].

### Environmental assessment

**Fig. 2:**
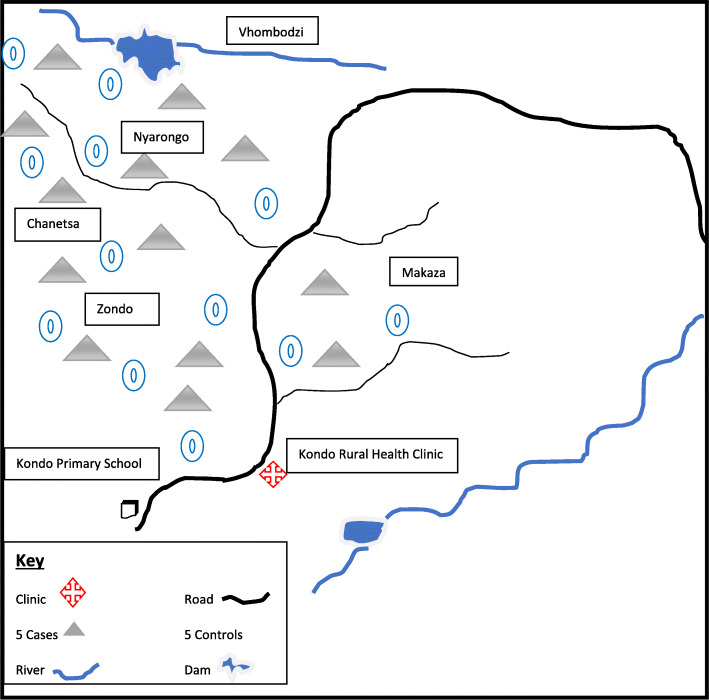
Distribution of malaria cases and controls in Mudzi district, Zimbabwe, 2019

The majority of the villages 60% (3/5) serviced by Kondo Rural Health Centre were located within 3 km from Chanetsa Dam. There are two dams one closer to Kondo Rural Health Centre clinic and another one on the boundary between Nyarongo and Vhombodzi. Small streams and swamps were common features within the villages with most homesteads within 200 m from these open water bodies (Fig. 2). There were tall uncut grass and stagnant water pools around most homesteads presenting mosquito breeding sites. *Anopheles gambiensi* and *Anopheles arabiensis* presence were identified from the sampled mosquitos and larvae. Thirty-nine percent (49/126) of visited homesteads reported not having undergone IRS as either the spraying team did not visit the homesteads as they are far away from the road or they came when no one was around at home. This was also confirmed by the respective village health workers. Seventy-five percent of visited homesteads had houses with open eaves exposing residents to mosquito bites.

### Evaluation of the malaria outbreak emergency, preparedness and response

The clinic staff managed to notify the district health executive (DHE) within 24 h of the upsurge in malaria cases and the outbreak was declared within 5 days. The emergency preparedness and response team (EPR) managed to mobilize adequate medications (coartemether tablets) for the clinic but had a limited supply of rapid diagnostic kits which were only dispatched to the clinic and none were distributed to the village health workers.

### Factors associated with contracting malaria

In the bivariate analysis, statistically significant risk factors for contracting malaria were living in a house with open eaves [OR = 8.7, 95% CI = (3.1–24.6)], having stagnant water bodies within 200 m from a homestead [OR = 7.5, 95% CI = (3.2–17.8)], engaging in outdoor activities before dawn and after dusk [OR = 6.3, 95% CI = (2.6–15.2)] and having evening meals outdoors at night [OR = 4.5, 95% CI = (2.1–9.9)]. Living in a house that was sprayed (IRS) [OR = 0.3, 95% CI = (0.2–0.7)], wearing long-sleeved clothes when outdoors at night [OR = 0.3, 95% CI = (0.2–0.7)] and having ever received health education on malaria [OR = 0.2, 95% CI = (0.1–0.6)] were significant protective factors against contracting malaria (Table 3).

**Table 3:** Factors associated with malaria infection in Mudzi district, Zimbabwe, 2019

Variable	Exposure Status	Cases *n* = 63 (%)	Controls *n* = 63 (%)	cOR (95% CI)	aOR (95% CI)
House with open eaves	Yes	58 (92)	36 (57)	8.7 (3.1–24.6) *	5.4 (1.2–23.3) **
	No	5 (8)	27 (43)		
Stagnant water body near house	Yes	54 (86)	28 (44)	7.5 (3.2–17.8) *	8.0 (2.3–28.6) **
	No	9 (14)	35 (56)		
Outdoor activities before dawn and after dusk	Yes	55 (87)	33 (52)	6.3 (2.6–15.2) *	8.3 (1.1–62.7) **
	No	8 (13)	30 (48)		
Evening meals	Outdoors	50 (79)	29 (46)	4.5 (2.1–9.9) *	2.4 (0.3–18.3)
	Indoors	13 (21)	34 (54)		
House Sprayed (IRS)	Yes	30 (48)	47 (75)	0.3 (0.2–0.7) *	0.4 (0.1–1.2)
	No	33 (52)	16 (25)		
Wearing long-sleeved clothes when outdoors	Yes	15 (24)	21 (33)	0.3 (0.2–0.7) *	0.2 (0.1–0.4) **
	No	48 (76)	42 (67)		
Ever received health education on malaria	Yes	47 (75)	59 (94)	0.2 (0.1–0.6) *	0.1 (0.0–0.9) **
	No	16 (25)	4 (6)		

*significant variable in bivariate analysis **significant variable in multivariate analysis

*cOR* crude odds ratio, *aOR* adjusted odds ratio

The association between engaging in outdoor activities before dawn and after dusk and contracting malaria infection stratified by sex were analysed. The crude odds ratio for engaging in outdoor activities before dawn and after dusk lies in between the stratum specific odds ratios. Therefore, the association between contracting malaria and engaging in outdoor activities before dawn and after dusk was modified by sex (male or female). Males who engaged in outdoor activities before dawn and after dusk were 7.3 times [OR = 7.3, 95% CI = (1.8–29.5)] likely to contract malaria compared to 5.4 times [OR = 5.4, 95% CI = (1.7–17.6)] among females who also engaged in outdoor activities before dawn and after dusk (Table 4).

**Table 4:** Relationship between engaging in outdoor activities and contracting malaria in Mudzi district, Zimbabwe stratified by sex

Variable	Category	Outdoor Activity	Cases	Controls	OR (95% CI)	*P*-Value
Sex	Male	Yes	22	16	7.3 (1.8–29.5)	0.01
		No	3	16		
	Female	Yes	33	17	5.4 (1.7–17.6)	0.01
		No	5	14		
Crude OR			55	33	6.3 (2.6–15.2)	< 0.001
			8	30		

In the multivariate analysis, a stagnant water body near a house [aOR = 8.0, 95%CI = (2.3–28.6)] and engaging in outdoor activities before dawn and after dusk [aOR = 8.3, 95%CI = (1.1–62.7)] were significant risk factors associated with contracting malaria in villages around Kondo Rural Health Centre adjusted for other factors. Wearing long-sleeved clothes when outdoors at night [aOR = 0.2, 95%CI = (0.1–0.4)] was a significant independent protective factor for contracting malaria (Table 3).

## Discussion

In our study, we found that there were a significant number of households that were not sprayed during the previous indoor residual spraying, stagnant water bodies around homesteads due to the rainy season, a lot of homesteads having houses with open eaves and the majority of families having evening meals outdoors and that malaria transmission was mainly due to outdoor activities and inadequate vector control.

The outbreak occurred following the rains that had poured in the area as evidenced by the increase in stagnant water bodies and tall uncut grass around homesteads which were potential mosquito breeding sites. The district had recorded an average of 245 mm of rainfall in December 2018 which increased to 565 mm in January 2019 with an average temperature of 28 Degrees Celsius [13]. This occurrence of an outbreak following the rains and characterised by stagnant water bodies near homes and tall grass is similar to findings by Muchena et al. in Shamva district were having long grass near the home had a 2.61 times risk of contracting malaria whilst having a body of water near the home had a 3.41 times risk of contracting malaria and they recommended larval source management to ensure few vector breeding sites [14].

The majority of cases were from Chanetsa and Nyarongo villages which were within 3 km from Chanetsa Dam and the area was swampy with more stagnant water bodies and this exposed the villagers to mosquito bites considering the majority of families had evening meals outdoors without putting on any protective clothing or use of topical mosquito repellents. This finding is similar to what has been reported by Chiruvu et al. in Beitbridge where staying 3 km away from a stagnant water body was 0.3 times protective from contracting malaria [15]. Failure to eliminate mosquito breeding sites by residents in malaria endemic areas has often resulted in malaria outbreaks and some studies in such areas have recommended the need for integrated and coordinated prevention and control measures to complement IRS [16, 17].

Kondo Rural Health Centre Clinic had been utilising village health workers in the diagnosis and treatment of uncomplicated malaria in the community before the outbreak and into the first and second week however as the epidemic progressed this could not be sustained due to a limited supply of RDT kits from the district. This could have been very effective especially in areas such as Vhombodzi and Nyarongo which were more than 10 km from the clinic given they had no readily available public transport to the health facility. This has been emphasized by Mutsigiri-Murewanhema et al. where they stated the need for VHWs to have malaria commodities at all times if their existence is to make a difference [18]. A longitudinal study in Zambia by Counihan et al. found that appropriately trained and supervised community health workers could use RDTs safely and accurately within the community for up to 12 months post-training [19].

The community had adequate health education on malaria however was not putting the knowledge into practice as characterised by the preventable mosquito breeding sites around houses such as stagnant water pools, tall uncut grass and failure to wear long-sleeved clothes when outdoors at night. This is in contrast to findings by Vundule and Mharakurwa in their study on knowledge, practices, and perceptions about malaria in rural communities were taking preventive measures was significantly related to knowledge of the causes of malaria [20].

A considerable number of community members were engaged in outdoor activities before dawn and after dusk without wearing long-sleeved clothes or using topical mosquito repellents exposing themselves to mosquito bites. This finding is similar to a study by Mugwagwa et al. in Honde valley were they found that engaging in outdoor activities before dawn and after dusk with considerable low utilization of mosquito repellents had a 2.1 times risk of contracting malaria [17].

The community demonstrated good health-seeking behaviour despite the majority of them being members of the apostolic sect as they managed to seek health care within 3 days of onset of illness and none reported use of alternative medicine for the treatment of malaria. This is in contrast to findings by Mugwagwa et al. in Honde Valley where the majority of the population were members of the apostolic sect and 38% of cases reported having used alternative medicine with the frequently mentioned being holy water [17].

The District’s EPR team responded swiftly to the outbreak but could not provide adequate RDT kits to village health workers who could have made a difference through active case finding and community treatment of the uncomplicated cases thereby reducing the malaria transmission rate. This is in contrast to findings by Kureya et al. in Chipinge where there were adequate supplies of RDT check kits and blood slides for laboratory investigation of malaria [21].

A limitation in our study was inadequate malaria diagnostic kits to confirm all enrolled controls to be malaria negative hence this misclassified some controls which might have been under the incubation period, therefore, being cases, which could then have underestimated our measures of association. However, despite these limitations’ meaningful inferences relevant to the study population could be drawn from our findings. This study strengthens the importance of complementary activities to indoor residual spraying in eliminating malaria morbidity and mortality in endemic areas as the implementation of these were successful in containing this outbreak.

## Conclusion

The majority of the malaria cases were from Makaza, Chanetsa and Nyarongo villages with the risk factors for contracting malaria being having a stagnant water pool near the homestead and engaging in outdoor activities before dawn and after dusk. Wearing long-sleeved clothing when outdoors at night was a significant protective factor against contracting malaria. The health facility had a well-prepared outbreak response plan with adequate medicines and but had a shortage of diagnostic kits. The community was knowledgeable about the causes and risk factors for malaria.

### Recommendations

Following our findings in this study, we recommended immediate social behaviour change communication by the health promotion officer on wearing long-sleeved clothes when outside at night. We also recommended immediate sourcing by the district medical officer and distribution by the district environmental health officer of RDT kits to village health workers. Village health workers were recommended to immediately commence community mobilisation on the destruction of mosquito breeding sites such as stagnant water bodies near homes and cutting tall grass around homesteads. Lastly, training of individuals drawn from the local community in IRS by the district environmental health officer was recommended before the next spraying schedule as utilisation of these cadres would ensure 100% coverage.

### Public health actions

The PHO was involved in active case finding during the research and assisted two young children with transport to the clinic. The PHO did an environmental assessment to identify mosquito breeding sites in the community and gave feedback to the VHWs to ensure the destruction of these sites. The PHO gave health education and awareness of malaria prevention methods at the end of every interview.

## Supplementary Information

**Additional file 1.**

## Data Availability

The datasets generated and analysed during the current study are available from the corresponding author on reasonable request.

## Abbreviations

ART: Antiretroviral Therapy
AIDS: Acquired Immunodeficiency Syndrome
DEHO: District Environmental Health Officer
DMO: District Medical Officer
EPR: Emergency Preparedness and Report
IDSR: Integrated Disease Surveillance and Report
IRS: Indoor Residual Spraying
LLITNs: Long Lasting Insecticide Treated Nets
MOHCC: Ministry of Health and Child Care
NMCP: National Malaria Control Program
PHO: Public Health Officer
PMD: Provincial Medical Director
RDT: Rapid Diagnostic Test
VHW: Village Health Worker
WHO/AFRO: World Health Organization African Region
