# Supplementary material for: Factors associated with malaria infection in Mudzi District, Mashonaland East Zimbabwe, 2019: a case-control study
Source: BMC Public Health. 2020 Nov 19;20:1745. doi: 10.1186/s12889-020-09872-2 (PMC7678088; doi:10.1186/s12889-020-09872-2)
Supplement: Supplementary file 1 — Additional file 1. [file 12889_2020_9872_MOESM1_ESM.docx]

**Malaria Knowledge Assessment**

The following questions were asked regards residents knowledge on malaria.

1. What do you think causes malaria?

Malaria parasite [ ] Bite by infected mosquito [ ] Witchcraft [ ] Infected fruits Dog bite [ ]

1. What are the signs and symptoms of malaria?

Fever [ ] Headache [ ] Shivering/Chills [ ] Joint pains [ ] Vomiting [ ]

1. What may increase someone’s risk of contracting malaria?

Staying out late at night [ ] Poor health [ ] Stagnant water near home [ ]

Contact with malaria patient [ ] Eating contaminated food [ ]

1. How can malaria be prevented?

Spraying insecticide [ ] Trimming bushes around the house [ ]

Wearing long clothes when outdoors [ ] Burning mosquito coil [ ]

Making fire and smoke [ ]

**Each question had five responses. Knowledge was then rated using a Likert scale as highlighted in the table below:**

| Score | Rating |
| --- | --- |
| 0-1 | Poor |
| 2-3 | Fair |
| 4-5 | Good |
